# Geometric morphometrics reveals shifts in flower shape symmetry and size following gene knockdown of *CYCLOIDEA* and *ANTHOCYANIDIN SYNTHASE*

**DOI:** 10.1186/s12870-017-1152-x

**Published:** 2017-11-17

**Authors:** Brent A. Berger, Vincent A. Ricigliano, Yoland Savriama, Aedric Lim, Veronica Thompson, Dianella G. Howarth

**Affiliations:** 1 Department of Biological Sciences, St. John’s University, 8000 Utopia Parkway, Queens, NY 11439 USA; 2 USDA-ARS, Carl Hayden Bee Research Center, 2000 E. Allen Road, Tucson, AZ 85719 USA; 3 Institute of Biotechnology, University of Helsinki, PO Box 56 (Viikinkaari 5), FI-00014 Helsinki, Finland

**Keywords:** Geometric morphometrics, Virus-induced gene silencing, *CYCLOIDEA*, *Fedia graciliflora*, Floral symmetry, *ANTHOCYANIDIN SYNTHASE*

## Abstract

**Background:**

While floral symmetry has traditionally been assessed qualitatively, recent advances in geometric morphometrics have opened up new avenues to specifically quantify flower shape and size using robust multivariate statistical methods. In this study, we examine, for the first time, the ability of geometric morphometrics to detect morphological differences in floral dorsoventral asymmetry following virus-induced gene silencing (VIGS). Using *Fedia graciliflora* Fisch. & Meyer (Valerianaceae) as a model, corolla shape of untreated flowers was compared using canonical variate analysis to knockdown phenotypes of *CYCLOIDEA2A* (*FgCYC2A*), *ANTHOCYANIDIN SYNTHASE* (*FgANS*), and empty vector controls*.*

**Results:**

Untreated flowers and all VIGS treatments were morphologically distinct from each other, suggesting that VIGS may cause subtle shifts in floral shape. Knockdowns of *FgCYC2A* were the most dramatic, affecting the position of dorsal petals in relation to lateral petals, thereby resulting in more actinomorphic-like flowers. Additionally, *FgANS* knockdowns developed larger flowers with wider corolla tube openings.

**Conclusions:**

These results provide a method to quantify the role that specific genes play in the developmental pathway affecting the dorsoventral axis of symmetry in zygomorphic flowers. Additionally, they suggest that *ANS* may have an unintended effect on floral size and shape.

**Electronic supplementary material:**

The online version of this article doi: (10.1186/s12870-017-1152-x) contains supplementary material, which is available to authorized users.

## Background

Natural diversity in gene expression, divergence, and function can now be examined in non-model organisms and across clades [1, 2] with the advent of new molecular tools and improved genetic resources (e.g., transcriptomes and an increasing number of genomes). In angiosperms, a growing number of non-model plants are emerging to characterise the role(s) of specific genes, or gene copies following polyploidization, in the evolution of morphological novelty, especially as it pertains to floral development (e.g., flexibility in floral structures in *Nigella* (Ranunculaceae) [3]; inflated calyx syndrome in *Physalis* (Solanaceae) [4]; and pollinator shifts in *Mimulus* (Phrymaceae) [5]). Examining gene function relies on assaying phenotypic shifts following experimental changes in the amount, location, or timing of candidate gene expression. Pinpointing precise phenotypic differences, however, can be challenging when the shift is subtle or due to redundant gene function. It can also be more difficult because of natural variation already present in the genomes of wild populations. Therefore, there is a need to be able to quantify fine-scale shape changes when using reverse genetics. Here, we use the non-model plant, *Fedia graciliflora* (Valerianaceae), to show the power of coupling reverse genetics with geometric morphometric techniques to quantify morphological variation.

**Fig. 1:**
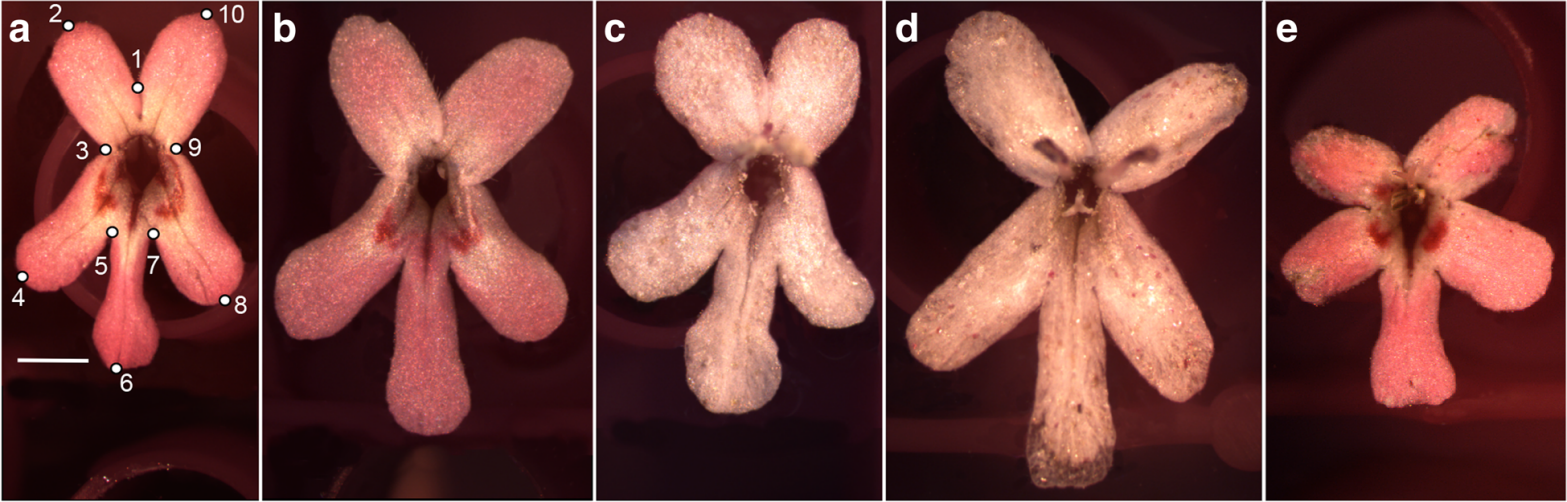
*Fedia graciliflora* flower phenotypes obtained through virus-induced gene silencing (VIGS). **a** Untreated flower with landmarks used to capture shape data. **b** Mock-treated (TRV2-E) control flower. **c** *F. graciliflora ANTHOCYANIDIN SYNTHASE* (*FgANS*) single knockdown flower. **d** *F. graciliflora CYCLOIDEA2A* (*FgCYC2A*) + *FgANS* double knockdown flower. **e** *F. graciliflora FgCYC2A* single knockdown flower. In all images, scale bar = 2 mm

Valerianaceae (Caprifoliaceae; Dipsacales) comprises seven genera (*c*. 350 species) and exhibits considerable diversity in floral form and fruit type [6, 7]. Prior molecular work provides strong evidence that at least three morphologically circumscribed genera are embedded within other lineages [8], and, thus, represent unique opportunities to study the evolution of novel characters and their underlying genetic mechanism(s). One such example, exemplified by the embedded genus *Fedia* Gaertn. (3 spp.) within *Valerianella* Mill. (*c*. 50 spp.), is the evolution of larger, strongly zygomorphic pink flowers with two ovate dorsal petals and marginal hairs, glabrous and elongated lateral and ventral petals developing distinct dark pink pollination guides, and elongated corolla tubes (Fig. 1a) [9] from a pseudo-actinomorphic, smaller white flowered lineage. Cytotaxonomic [10] and genome size [11] studies reveal that *Fedia* likely evolved as the result of polyploidization (*x* = 8; 2n = 32; 2C = 1.14–1.79), possessing almost twice as much genomic content based on DNA flow cytometry compared to *Valerianella* (*x* = 8; 2n = 16; 2C = 0.39–0.61; [11]). Examination of 10 native populations of *Fedia graciliflora* Fisch. & C.A. Mey. across the Mediterranean revealed diploid chromosome numbers were identical with a single hexaploid exception (2n = 48); however, chromosome structure indicates that translocation, (i.e., the breaking and reattaching of two non-homologous chromosomes) is a common phenomenon [10], a finding also observed in *Arabidopsis* populations [12], which partially explains the high amount of within population variation. Collectively, the recent evolution of morphological characters including strongly zygomorphic flowers, plus the dynamic genomic structure of *Fedia*, make this a valuable genus to study the molecular mechanism(s) of phenotypic transitions.

One phenotype often associated with plant diversification and adaptive evolution is floral symmetry [13–15], with shifts to zygomorphy (i.e., bilaterally symmetrical flowers) [16] considered more specialised due to correlations with increased speciation rates [17] likely associated with changes in pollination syndrome [18]. Shifts from actinomorphic to zygomorphic flowers have repeatedly been shown to involve recruitment of several transcription factors, with the greatest focus on the ECE clade of TCP genes (i.e., *CYCLOIDEA* (*CYC*) and *DICHOTOMA* (*DICH*) in the CYC2 clade [19, 20]). Examination of multiple independent shifts to zygomorphy reveals expression of *CYC*-like genes from the ECE clade [20] is restricted to the dorsal region (i.e., toward the upper petals) of the corolla (see [21, 22]) with loss-of-function mutants of *CYC*-like genes resulting in more radial-like flowers occurring in both asterids and rosids [19, 23–25]. Additionally, duplications of *CYC*-like genes often correlate with shifts to bilateral symmetry [15, 26]. In *Antirrhinum*, both CYC2 gene clade members have redundant function, and the loss-of-function of both copies in parallel is necessary to generate a fully radial flower. *Fedia graciliflora* possesses two paralogs of the CYC2 gene clade, *CYC2A* and *CYC2B*, resulting from a duplication around the divergence of the bilaterally symmetrical Caprifoliaceae from the radially symmetrical Adoxaceae [27]. The general expression patterns of *CYC2* genes are that *CYC2B* is expressed in both dorsal and lateral petals, while *CYC2A* becomes more restricted to the dorsal petals [28]. Given this general pattern, we hypothesize that knocking down a single *CYC2*-like copy will result in a partial shift to radial symmetry in *F. graciliflora*.

Because loss-of-function mutants are not readily available for most non-model plants, we utilise virus-induced gene silencing (VIGS) to assay gene function. The technique has increasingly been used in evolutionary developmental biology (evo-devo) studies (e.g., [3, 5, 25, 29–36] to downregulate a number of protein coding genes implicated in floral development where stable transformation protocols are not available [37–39]. VIGS facilitates the downregulation of either individual or multiple genes simultaneously [29, 39–41]; and, recently, it has been shown capable of knocking down epigenetic modifiers involved in RNA-directed DNA methylation in *Arabidopsis* [42]. Additionally, VIGS is useful for studying the regulation of plant growth and differentiation originating in the meristem and floral organs (e.g., [43, 44]), and, unless targeted, it does not compromise fertility [45]. VIGS generally results in mosaic phenotypes with large amounts of variation across knockdowns [39–41, 46, 47]. In examining floral shape and symmetry, interpreting these variable results can be complex; thus, a reporter gene is often simultaneously knocked down in conjunction with the gene(s) of interest (GOI) to verify successful inoculation, and presumably downregulation [30, 37, 48]. One often targeted reporter gene in floral tissues is *ANTHOCYANIDIN SYNTHASE* (*ANS;* [3, 37, 49, 50]), which is an enzyme that plays a key biochemical role in each of the three major anthocyanin pathways of land plants [51]. VIGS of *ANS* should result in unpigmented floral tissue due to the downregulation of the anthocyanin pathway without altering floral shape (see Fig. 1; Additional file 1). Even when using discernable reporter genes, however, a large amount of natural variation and subtle shape changes can still make interpretation difficult. For this reason, we incorporate geometric morphometrics (e.g., [52–54]) to more precisely quantify and visualise morphological variation in size and shape, including patterns of symmetry [55–61].

Here, for the first time, we couple geometric morphometrics with VIGS in plants to statistically analyse shape and symmetry changes that result from knocking down the *CYCLOIDEA2A*-like (*CYC2A*) ortholog of the CYC2 clade [20] in *Fedia graciliflora*. We quantify and compare floral shape and size data from untreated (Fig. 1a), mock-treated (empty TRV2) control plants (TRV2-E; Fig. 1b), *ANS* knockdowns (TRV2-*FgANS*; Fig. 1c), double *CYC2A* and *ANS* (TRV2-*FgCYC2A* + TRV2-*FgANS*; Fig. 1d) knockdowns, and single *CYC2A* (TRV2-*FgCYC2A*; Fig. 1e) knockdowns to quantify and characterise potential shape changes in loss of function *FgCYC2A* and *FgANS* plants. Our findings of consistent shifts in petal position of the corolla corroborate similar results in other plants (e.g., *Antirrhinum* [19] and *Pisum* [25]) that a loss of function of a *CYC2A*-like ortholog results in a more radially symmetrical flower. We also show that *FgANS* knockdowns confer subtle changes in floral shape and size, especially with regard to length of the dorsal petals and the size of the corolla tube opening, suggesting the enzyme may be directly or indirectly involved in flower growth and development.

## Methods

### Plant growing techniques

Seeds of *Fedia graciliflora* Fisch. & C.A. Mey. were purchased from Malta Wild Plants (www.maltawildplants.com). All seeds were cold stratified at 4^°^ C prior to surface sterilization and subsequent germination using standard methods. Seedlings were transplanted to individual pots (4 × 4 × 4 cm) and grown under 117 W of full spectrum (6500 K) T5 florescent lights at 20–22^°^ C for 3–4 weeks to promote vegetative growth. To initiate flowering, 39 W of full spectrum bulbs were replaced with red light spectrum (2700 K) bulbs on each shelf of the growth chamber. *FgANS* and *FgCYC2A* knockdown plants were inoculated with recombinant *Agrobacterium tumefaciens* (*A. tumefaciens*) cultures between 2 and 4 weeks after red light conditions were implemented and at the onset of inflorescence development.

### Cloning *FgANS* and *FgCYC2A*

Total RNA was isolated from flash-frozen, pooled *F. graciliflora* floral bud samples (i.e., buds collected at the onset of flowering through pre-anthesis) using an RNeasy Plant Mini Kit (Qiagen, Valencia, CA) following the manufacturer’s protocol, and including the on-column RNase-Free DNaseI step (Qiagen, Valencia, CA). cDNA was generated from 2 μL of total RNA using the Superscript III One Step RT-PCR kit with Platinum *Taq* (Invitrogen, Alameda, CA). The *FgANS* gene was cloned using degenerate primers (Additional file 2) based on GenBank accessions of *Chrysanthemum* × *morifolium* (Asteraceae; EU810810), *Ipomoea hederifolia* (Convolvulaceae; AB618110), *Gerbera* hybrid cultivar Tacora (Asteraceae; AY997840), *Dahlia pinnata* (Asteraceae; AB591830), and *Lactuca sativa* (Asteraceae; AB525912). The *FgCYC2A* gene was cloned as previously described [27]. Conserved, sequence specific primers (Additional file 2) were used to generate primary amplicons of *FgANS* and *FgCYC2A*, which were re-amplified using appended sequence specific primers including XbaI and BamHI restriction sites (Additional files 2 and 3). The full-length sequence of *FgCYC2A* was amplified using 5′ and 3′ RACE (Rapid Amplification of cDNA Ends; SMARTer RACE 5′/3′ Kit, Clontech; Additional file 2) for the development of primers in regions lacking sequence similarity with other *CYC*-like paralogs. All PCR products were sequenced, verified using BLAST (NCBI), and deposited in GenBank (KX981057 and KX981058).

### Viral inoculations

Tobacco rattle virus (TRV) binary expression vectors containing RNA1 and RNA2 [62, 63] were generated by ligating antisense fragments of *FgANS* (455 bp) and *FgCYC2A* (296 bp) into the pTRV2 binary vector to generate pTRV2-*FgANS* and pTRV2-*FgCYC2A* recombinant vectors, respectively (Additional file 3).

Recombinant pTRV2 constructs were introduced into *A. tumefaciens* (strain EHA105) (ATCC) via the freeze-thaw method and maintained on LB media containing 25 mg L^−1^ rifampicin (selection for EHA105) and 50 mg L^−1^ kanamycin (selection for binary vector). Liquid cultures were initiated from LB selection plates by subculture to 5 mL of LB selection broth. These cultures were incubated at 28 °C for 48–72 h with shaking, after which they were inoculated into 30 mL LB containing 200 μM acetosyringone and incubated for 12–24 h, resuspended in infiltration media (10 mM MgCl_2_, 10 mM MES, 200 μM acetosyringone) to an optical density of 0.8–1.0, and incubated at 25 °C for 2 h [63]. Co-infiltration of *A. tumefaciens* suspension containing pTRV1 with pTRV2-E (1: 1), pTRV2-*FgANS* (1: 1), pTRV2-*FgCYC2A* (1: 1), or pTRV2-*FgCYC2A +* pTRV2-*FgANS* (1: 1: 1) was inoculated into the two sets of leaves immediately subtending developing inflorescences using a needleless syringe to increase viral load as previously described [62]. In addition to 14 untreated plants, 56 plants were inoculated as follows: 6 TRV2-E, 15 TRV2-*FgANS*, 8 TRV2-*FgCYC2A*, and 27 TRV2-*FgCYC2A* + TRV2-*FgANS*. Plants were left in a humidity chamber overnight for co-cultivation after which they were returned to the growth chamber.

### Quantifying expression and downregulation of *FgANS* and *FgCYC2A*

**Fig. 2:**
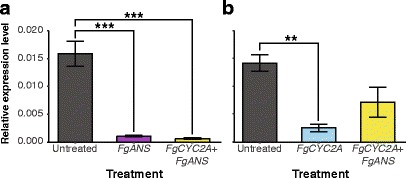
Quantitative real-time PCR (qPCR) analysis of *FgANS* and *FgCYC2A* in untreated samples and knockdown treatments. **a** qPCR of *FgANS* showing relative abundance levels in untreated samples (*N* = 4) compared to those in *FgANS* (*N* = 6) and *FgCYC2A + FgANS* (*N* = 3) knockdown flower buds. **b** qPCR of *FgCYC2A* showing relative abundance levels in untreated samples (*N* = 3) compared to those in *FgCYC2A* (*N* = 3) and *FgCYC2A* + *FgANS* (*N* = 3) knockdown flower buds. Mean and standard error bars for biological replicates are shown with statistical significance between treatments designated as follows: ***, *P* < 0.001, **, *P* < 0.01

Buds from each treatment were examined and collected 2–3 weeks post inoculation. Six biological replicates, each representing an independent inoculation, were obtained for each treatment, including untreated, and flash frozen in liquid nitrogen prior to total RNA extraction following the protocol previously mentioned. Buds from TRV2-*FgANS* and TRV2-*FgCYC2A +* TRV2-*FgANS* inoculated plants were preferentially selected based on their downregulated phenotype, while those inoculated solely with TRV2-*FgCYC2A* were sampled randomly. Primers were developed to amplify fragments of *FgANS* (360 bp) and *FgCYC2A* (169 bp) (Additional file 3) for quantitative real-time PCR (qPCR) analysis (Fig. 2; Additional file 4). PCR products were sequenced and BLAST verified (NCBI). RNA samples were normalised to 2 ng mL^−1^ and qPCR was performed using the iScript One-Step RT-PCR kit with SYBR Green (Bio-Rad, Hercules, CA). Relative expression levels of *FgANS*, *FgCYC2A*, *FgCYC2A + FgANS,* and the reference gene, *GLYCERALDEHYDE 3-PHOSPHATE DEHYDROGENASE* (*GAPDH*), were calculated for three biological replicates run in duplicate (two technical replicates) using the 2^-ΔΔCT^ method [64]. Assays of *FgANS*, *FgCYC2A*, *FgCYC2A + FgANS,* and the reference gene were conducted in 96-well plates on a Bio-Rad MyIQ Single Color Real-Time PCR Detection System (Bio-Rad, Hercules, CA).

### Geometric morphometric analysis of symmetry

Here, we test the hypothesis that resultant phenotypes of *F. graciliflora* generated by VIGS knockdown of *FgCYC2A* and *FgCYC2A + FgANS* develop distinct morphologies in terms of shape and size compared to knockdowns of *FgANS*, mock-treated (TRV2-E) controls, and untreated controls. We used geometric morphometric methods combined with multivariate statistical shape analysis to test for differences between untreated and different knockdown floral phenotypes (e.g., [65, 66]). One hundred thirty-six flowers from a total of 70 plants were photographed in an identical manner using a Lumenara camera (Model # Infinity2-1C-ACS) mounted to a Zeiss Stemi-2000-C stereomicroscope. Given the mosaic nature of VIGS, flowers developing a whitish corolla tube (untreated is deep maroon) were randomly selected from plants in which *FgANS* was knocked down. Flowers from untreated, TRV2-E, and TRV2-*FgCYC2A* were all chosen at random. Upon removal from the inflorescence, reproductive structures of each flower were cut off and the flower was placed petals up in water. Prior to placing landmarks, images were reviewed and the following criteria was used in selecting the final image dataset: (1) flowers represented fully mature and open flowers; (2) no portion of the flower was submerged in water or folded over; and (3) the location of landmarks was devoid of overlapping reproductive structures that may affect reproducibility in landmark placement. For each photograph, a suite of 10 landmarks was collected in two dimensions using tpsDig2 version 2.17 [67] to characterise the architecture of *F. graciliflora* flowers (Fig. 1a). These landmarks were located either at the points of intersection between primary and secondary veins with the petal margin or at the junction of petal bases.

*Fedia graciliflora* flowers possess an axis of bilateral symmetry that runs between the dorsal petals through the middle of the ventral petal (described by landmarks 1 and 6, Fig. 1a), and which was taken into account with the method of object symmetry [56–58, 60]. Following this approach, the original configuration of landmarks is populated by paired landmarks that are mirror images of each other relative to the axis of symmetry and are located outside of it, while unpaired landmarks lie on the axis of symmetry. First, the original configurations of landmarks are duplicated, and then, these copies are reflected with an appropriate relabelling of the paired landmarks. Thereafter, a Generalized Procrustes fit is applied to the doubled dataset and removes extraneous information of size, location, and orientation, to extract shape data according to a least squares criterion. A mean shape configuration (consensus) is computed and variation around this mean (e.g., [65, 68–70]) is decomposed into biologically meaningful components. Because we are interested in differences between the untreated flowers and phenotypes of all treatments at the population level, we only consider the component of symmetric shape variation, which is computed by averaging the Procrustes coordinates for the original and appropriately transformed and relabelled copies of the landmarks of each individual. Consequently, the two-way mixed model Procrustes analysis of variance (ANOVA) traditionally used in studies of bilaterally symmetric structures is simplified here and the total shape variation is decomposed according to the main effect of ‘individual’ and measurement error due to imaging and digitising [58, 60, 71, 72]. To test for measurement error due to imaging and digitising, each flower was photographed twice and each picture was digitised twice.

Centroid size, the most common and explicit measure of size in geometric morphometrics, was computed as the square root of the sum of the squared distances of all landmarks from their centroid (e.g., [65, 73, 74]). A one-way ANOVA was used with centroid size to test for differences in size between the untreated flowers and all other treatments (Additional file 5). The statistical significance of pairwise differences in size was assessed via Tukey’s ‘Honest Significant Difference’ (HSD) with adjustment for the multiple comparisons as implemented in R [75] (Additional file 5). To avoid potential confounding effects among groups due to size in our analyses, we used a pooled within-group multivariate regression of the Procrustes coordinates on centroid size [76]. The statistical significance of the relationship between the shape variables and the centroid sizes was assessed by a permutation test with 10,000 rounds of random permutations [76, 77]. There was a significant effect of size on shape (*P-*value = 0.002) and the regression of shape on centroid size accounted for 3.6% of total shape variation.

To test for differences in shape and to visualize the patterns of variation between the untreated controls and knockdown treatments, we used a canonical variate analysis (CVA). This is a discriminant analysis designed to maximize variation among groups and minimize variation within groups to obtain the best possible segregation among groups (e.g., [78–82]). Since there was a significant effect of size on shape, we used only the residual component of the regression of shape on centroid size for the CVA that is separated from the allometric component of within-group variation and size-related differences between groups. The statistical significance of pairwise differences in mean shapes was assessed by a permutation test using Procrustes distance (e.g., [53, 65, 70]), which is the standard metric in landmark based methods describing a measure of morphological difference among groups (e.g., [83]) (Additional file 6). We also used a discriminant analysis to make specific pairwise comparisons between the average overall shape of untreated flowers compared to the mean shape of each treatment to visualise the average shape departure of each group compared to the untreated samples. All analyses were carried out using MorphoJ [84] and R [75].

## Results

### Knockdown of *FgANS* and *FgCYC2A* genes

To characterise putative shape changes resulting from loss-of-function of *FgCYC2A* and *FgANS*, we developed the tobacco rattle virus (TRV)-mediated VIGS system [62, 85] in *F. graciliflora*. Binary vector constructs (pTRV1 and pTRV2) were introduced into *A. tumefaciens* and were used to express antisense fragments against the target gene transcripts. Our strategy was three-fold: (1) to develop an efficient RNA-silencing system specific to *F. graciliflora;* (2) to apply this system to examine the putative role of *FgCYC2A* in *F. graciliflora* floral development; and (3) to characterise resultant mutant phenotypes using geometric morphometric analysis. To utilise VIGS in *F. graciliflora*, an antisense fragment of *FgANS* was placed under the constitutive control of a double CaMV35S promoter (TRV2-*FgANS*; Additional file 3). Although several means of inoculation were attempted, including vacuum infiltration and floral dip, direct needleless syringe infiltration through the underside of leaves immediately preceding the developing inflorescences yielded the most consistent downregulation of *FgANS* (the same was observed for *FgCYC2A*).

Of the 42 plants inoculated with TRV2-*FgANS* or a combination of TRV2-*CYC2A* + TRV2-*FgANS*, all 42 plants produced at least some flowers exhibiting reduced levels of *ANS*. As previously observed [3, 37, 49, 50], VIGS of the *ANS* ortholog resulted in mosaic phenotypes, and the degree of downregulation varied from mild to strong, ranging from mostly white flowers with scattered anthocyanin-filled cells to splotchy anthocyanin-filled areas to completely downregulated white flowers (Additional file 1). Mock-treated plants (TRV2-E) were also compared to examine any possible effect that *A. tumefaciens* infiltration and subsequent systemic movement of the viral constructs into the meristem had on overall floral morphology.

Quantitative real-time PCR (qPCR) expression analysis was carried out on floral buds obtained from untreated controls (*N* = 7), TRV2-*FgANS* (*N* = 6), TRV2-*FgCYC2A* (*N* = 3), and TRV2-*FgCYC2A* + TRV2-*FgANS* (N = 3) treated plants to confirm downregulation was due to the knockdown of endogenous transcripts by viral inoculants (Fig. 2). No difference was detected between untreated and TRV2-E flowers (Additional files 7 and 8); however, significant reduction in *FgANS* transcript abundance was observed when comparing untreated levels to those detected in either TRV2-*FgANS* (*P* < 0.001) or TRV2-*FgCYC2A* + TRV2-*FgANS* (*P* < 0.001) knockdowns (Fig. 2a). To ascertain that the observed phenotypic variability of the corolla was due to knockdown of *FgCYC2A*, we compared levels of endogenous *FgCYC2A* transcript abundance in untreated buds with TRV2-*FgCYC2A* single knockdowns and TRV2-*FgCYC2A* + TRV2-*FgANS* knockdowns (Fig. 2b). A non-significant decrease in endogenous transcript levels was found in TRV2-*FgCYC2A* + TRV2-*FgANS* plants compared to untreated samples, while a significant decrease (*P* < 0.01) in endogenous transcript levels was observed between the untreated and TRV2-*FgCYC2A* plants (Fig. 2).

### Geometric morphometric analyses of VIGS knockdowns

ANOVAs for measurement error in the untreated samples reveal that the ‘individual’ main effect is significant, which means that the variation among flowers greatly exceeds the measurement error due to imaging (Additional file 9). ‘Imaging’ error represents the variation due to measurement error in taking pictures of the same individual in separate sessions. This term is significant, which means that the imaging error is larger than the error due to digitising. ‘Digitising’ error is the variation due to measurement error in digitising the same picture of the same individual in separate sessions. These results suggest that the biological variation at the population level largely exceeds all sources of measurement error due to imaging and digitising in the untreated sample. Similarly, measurement error is also negligible in all analyses involving other treatments.

**Fig. 3:**
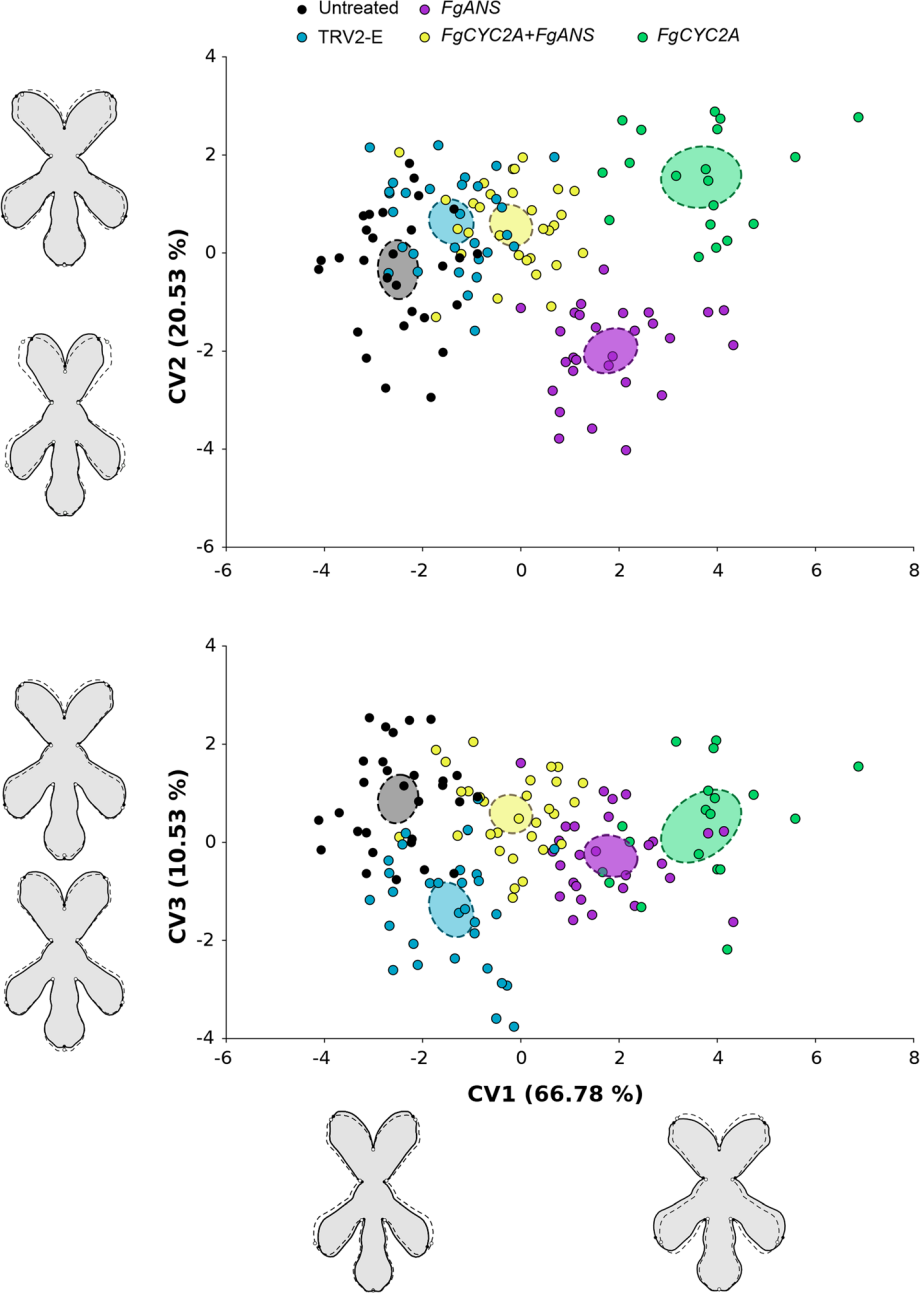
Canonical variate analysis (CVA) plots of *Fedia graciliflora* flower shape data. CVA is corrected for within-group allometry with mock-treated controls (TRV2-E) (*N* = 30), *FgANS* (*N* = 28), *FgCYC2A + FgANS* (N = 30), and *FgCYC2A* (*N* = 19) knockdown treatments compared to untreated (*N* = 29). 95% confidence ellipses of means for each group is represented (filled ellipses with dashed contours). The outline drawings of flowers show shape changes associated with each CV from the overall average shape (dotted outline and open circles) for CV1 scores of −3 and +4, for CV2 scores of −3 and +2, and for CV3 scores of −2 and +2 (solid black outline with grey background and solid black circles). Note that these outline drawings are an interpolated form of presentation from the actual landmarks based on the thin-plate spline technique that makes it easier to visualise shape changes. This means that the relevant information in this CVA is from the positions of the landmarks, not from the outline drawings

CVA plots display the patterns of morphological segregation for the component of symmetric shape variation among different groups (Fig. 3). CV1 accounts for 66.78%, CV2 for 20.53%, and CV3 for 10.53% of the amount of relative between-group variation. CV1 displays shape changes that primarily affect the width of the corolla tube and the location of dorsal and lateral petals. Specifically, from the negative to the positive direction, the landmarks placed at the tips of dorsal petals move downward and slightly away from the axis of symmetry, while the landmarks at the tips of the lateral petals move upward and slightly away from the axis of symmetry. CV1 separates all treatments from each other, with the greatest variation occurring between untreated samples and *FgCYC2A* knockdowns. Of note, both single gene knockdown plants of *FgANS* and *FgCYC2A* showed more of a shift from the negative to positive direction in CV1 than the *FgCYC2A + FgANS* double knockdown plants. CV2 represents shape changes that shift dorsal and lateral petals away from the axis of symmetry, with only minimal change along the dorsoventral axis. CV2 most clearly separates *FgCYC2A* from *FgANS* knockdowns, with dorsal and lateral petals closer to the axis of symmetry in *FgANS* and farther from the axis of symmetry in *FgCYC2A*. Collectively, CV1 and CV2 describe morphological change from zygomorphic to actinomorphic-like flowers. CV3 describes morphological differences that are mainly localised to the ventral region of flowers, but also reveals subtle changes to the dorsal region with mock-treated (TRV2-E) flowers having shorter ventral petals than the untreated controls (Fig. 3).

**Fig. 4:**
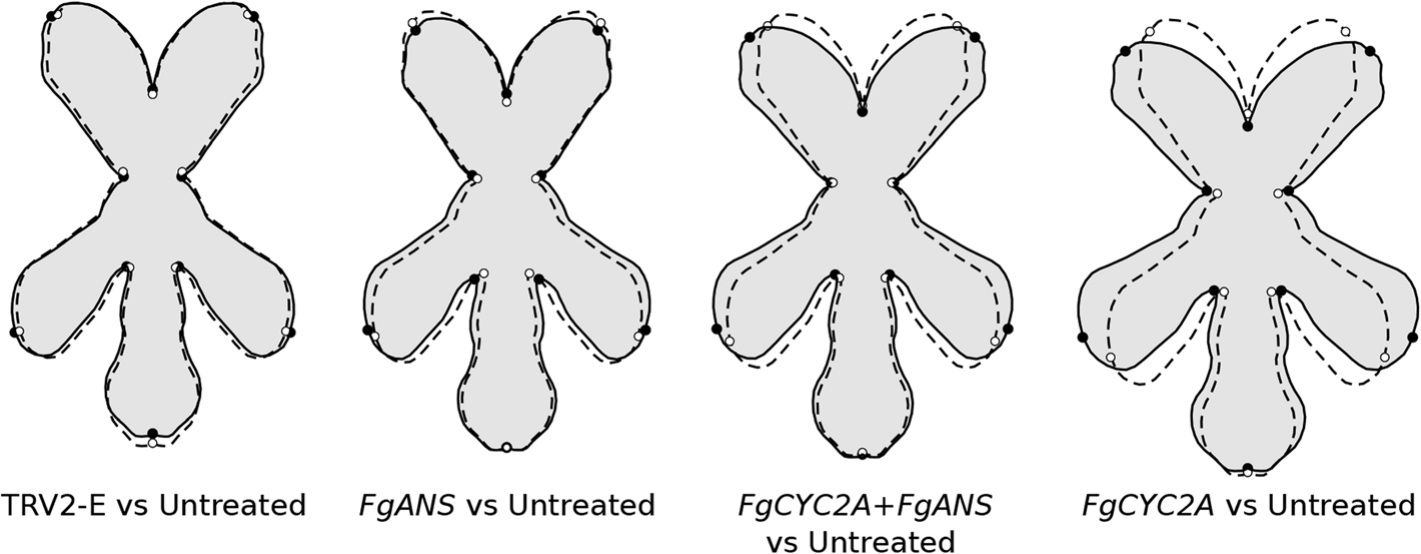
Pairwise comparisons of the untreated samples versus each treatment obtained via a discriminant analysis. The outline drawings of flowers show the shape change deviations from the untreated average shape (dotted outline and open circles) for each treatment (solid black outline with grey background and solid black circles) for a Procrustes distance of +0.1. Note that these outline drawings are an interpolated form of presentation from the actual landmarks based on the thin-plate spline technique that makes it easier to visualise shape changes. This means that the relevant information is from the positions of the landmarks, not from the outline drawings

**Fig. 5:**
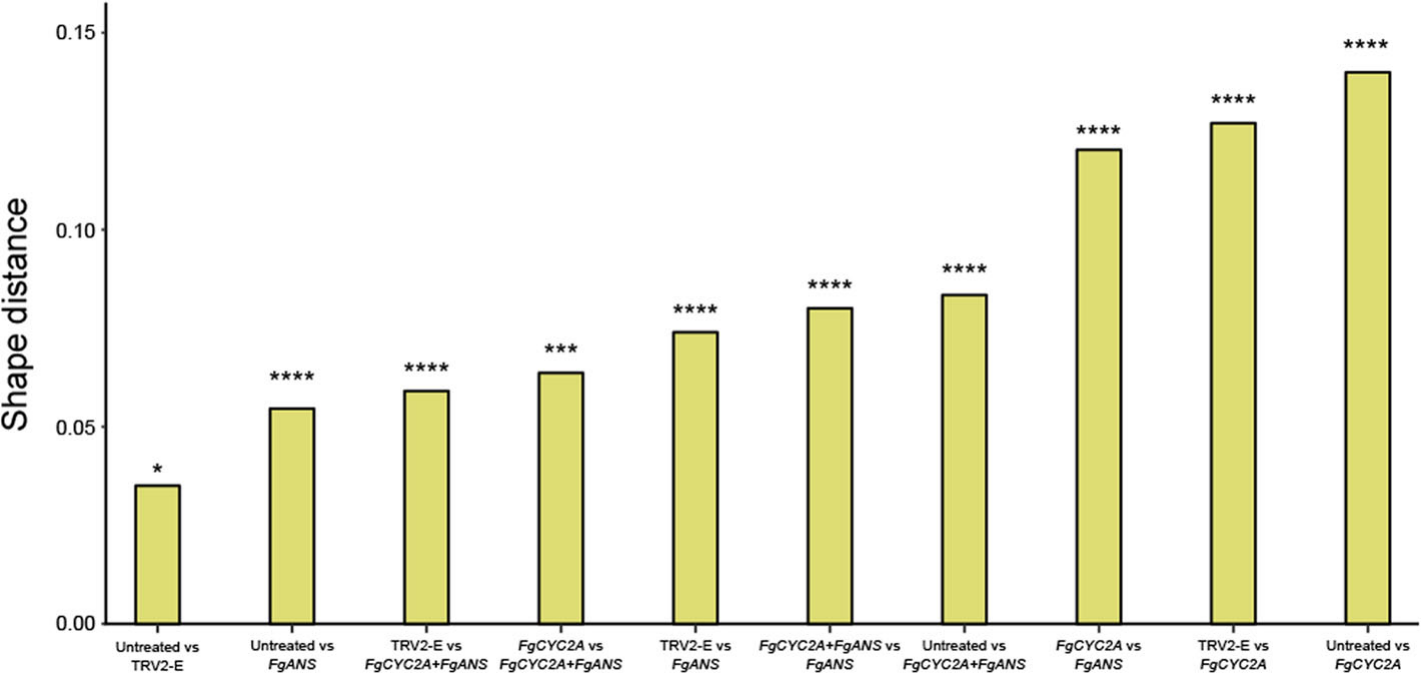
Procrustes distances among all possible pairs of groups. *P*-values for all pairwise tests are presented as follows: ****, *P* < 0.0001; ***, *P* < 0.001; *, *P* < 0.05

Pairwise comparisons made between untreated samples and each treatment (Fig. 4) reveals that flowers from TRV2-E plants were very similar to uninoculated flowers, with subtle differences in petal lengths. Furthermore, *FgANS* knockdown plants were also similar in overall shape to untreated ones, but with wider corolla tubes, shorter dorsal petals, and lateral petals shifted slightly toward the dorsal petals. Although flowers from *FgCYC2A* knockdowns were more extreme, both *FgCYC2A* and *FgCYC2A + FgANS* knockdowns demonstrated similar changes in morphology, with a definite shift toward a more radially symmetrical flower. Specifically, dorsal and lateral petals both lengthened and moved toward each other, decreasing the angle between them. Non-parametric tests reveal that all pairwise comparisons made among these groups are highly significant (Fig. 5, Additional file 6), which suggests that phenotypes from different knockdown treatments, as well as untreated flowers, are distinct from each other, with the largest morphological difference in petal position relative to the axis of bilateral symmetry exhibited by *FgCYC2A* knockdowns compared to untreated controls.

**Fig. 6:**
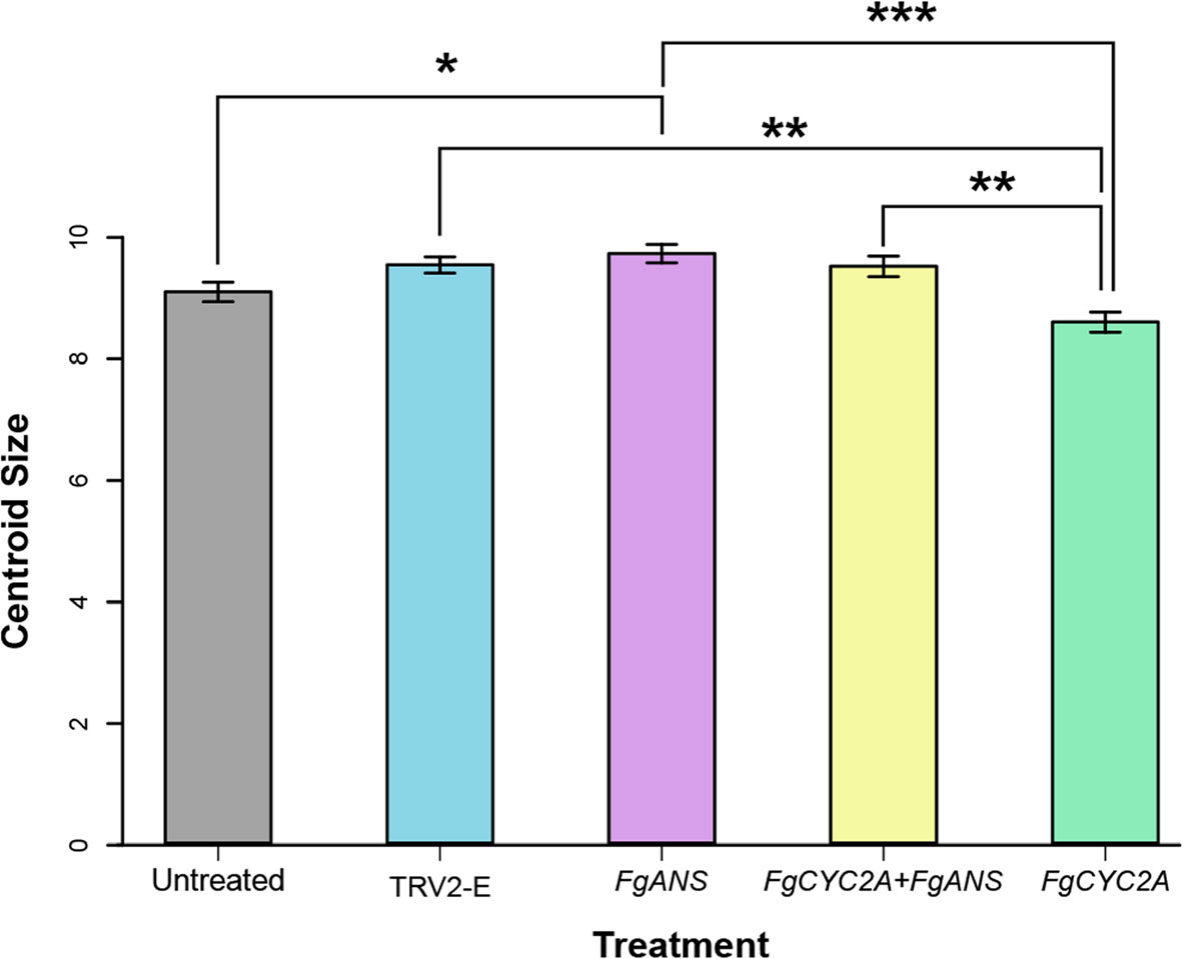
Tukey’s ‘HSD’ test for pairwise differences in centroid size between untreated flowers and other treatments with 95% family-wise confidence level. Mean and standard error bars are shown with statistical significance between treatments designated as follows: ***, *P* < 0.001; **, *P <* 0.01; *, *P* < 0.05

The ANOVA for centroid size concerning the comparisons among treatments suggests that there is a difference in size between untreated controls and all knockdown treatments. The statistical significance of pairwise differences in size was further assessed via Tukey’s ‘HSD’ with adjustment for multiple comparisons and indicates that there are significant differences in size between untreated flowers and *FgANS* knockdowns, with the latter being slightly larger, between TRV2-E and *FgCYC2A* knockdowns, between *FgANS* and *FgCYC2A* knockdowns, and between *FgCYC2A* + *FgANS* and *FgCYC2A* knockdowns, with *FgCYC2A* knockdown flowers being slightly smaller than all other treatments, including the untreated controls (Fig. 6, Additional file 5).

## Discussion

### Significant variation within knockdown flowers of *Fedia graciliflora*

As the number of tools for examining gene sequence, expression, and function continues to expand, the ability to utilise non-model species to better understand molecular processes is also becoming more tangible. These non-model species and clades provide a broad spectrum of natural variation to examine how changes in molecular sequence ultimately can shift morphology [26]. The ability to examine gene expression and function across a more diverse array of species can allow, for instance, comparisons of multiple iterations of the same morphological shift [86], or stepwise changes in morphology [28, 87–89].

VIGS is a powerful technique for examining knockdown phenotypes in non-model plants that is less labor intensive and more rapid than stable transformation approaches [40]. In *Fedia graciliflora*, for instance, it took an average of 3–4 weeks post inoculation to observe a downregulated phenotype. Unfortunately, VIGS phenotypes can be difficult to assay given that the knockdown of a GOI is often mosaic and transient [39–41, 46, 47]. To further complicate the assessment of VIGS knockdown phenotypes, inherent and heritable natural variation within recently evolved species can be difficult to tease apart. Despite the possible genomic background diversity in *Fedia*, it still poses as a very useful group for developmental studies of floral symmetry as each of the three species has strongly zygomorphic flowers with distinct dorsoventral arrangement of the petals. Additionally, *F. graciliflora* is fast growing and produces upwards of 100 flowers per plant.

### Quantifying shape change in *FgCYC2A* knockdowns

The role of *CYC2*-like genes in patterning floral symmetry has been examined across angiosperms, with much of the focus on the crown group of flowering plants, the core eudicots (see [21, 26]). Across core eudicots, including Dipsacales, the expression of CYC2 paralogs are dorsally restricted in bilaterally symmetrical flowers [21, 90]. We have previously shown that the degree of dorsal restriction is correlated with the differentiation between the dorsal and ventral regions (i.e., the degree of zygomorphy) in other Dipsacales species [22, 28]. In both asterid and rosid clades, loss-of-function of one or more CYC2 paralogs results in a shift toward a more radially symmetrical, ventralised flower [19, 23, 25]. In *Antirrhinum*, both CYC2 paralogs, *CYC* and *DICH*, must simultaneously exhibit a loss of function to generate a fully radial flower [19]. The loss of function of each paralog separately results in only a partial loss of bilateral symmetry, with *CYC* mutants conferring a stronger phenotype than *DICH* mutants [19], indicating the two paralogs have evolved a level of functional redundancy.

Along with the dorsal restriction of CYC2 paralogs, zygomorphic lineages tend to maintain two or more copies of *CYC2*-like genes (see [21, 26]). In Dipsacales, a duplication occurred around the diversification of the zygomorphic Caprifoliaceae *s.l.* resulting in two *CYC2*-like copies (*DipsCYC2A* and *DipsCYC2B*), both of which have orthologs (or additional paralogs through subsequent duplications) that are retained in most lineages, including *F. graciliflora* (i.e., *FgCYC2A* and *FgCYC2B*) [22, 27]. Therefore, by knocking down a single CYC2 paralog in *F. graciliflora*, *FgCYC2A*, we expect a partial loss of bilateral symmetry.

In fact, given the variable flowers of *F. graciliflora*, the mosaic nature of VIGS, and the functional redundancy of *CYC2*-like gene copies, it was difficult to consistently determine a knockdown phenotype by eye alone. However, by utilising geometric morphometrics to quantify shape change from photographs of flowers chosen randomly from each of the treatments, the shift toward a radially symmetrical flower was evident in both *FgCYC2A* and *FgCYC2A* + *FgANS* knockdowns compared to other treatments, as described by changes in CV1 and CV2 (Fig. 3) and in pairwise comparisons (Fig. 4).

Typical untreated flowers of *F. graciliflora* have an elongated ventral petal, that is often weakly clawed and lateral petals that are typically non-clawed and slightly shorter (Fig. 1a). Both ventral and lateral petals have thick, dark pink bands of anthocyanin pigment near the corolla opening serving as pollination guides. Dorsal petals, on the other hand, are shorter and often more rounded, and always lack additional pink markings. In contrast to untreated flowers, *FgCYC2A* knockdowns highlighted the expected phenotype, with a partial shift toward a more radially symmetrical flower. Lateral and dorsal petals appeared more ventralised, with both developing longer petals on average in comparison to untreated ones. Dorsal petals also often contained anthocyanin markings, similar to ventral and lateral petals. The location of petals shifted away from the dorsoventral axis into more radial positions as well, with dorsal petals shifting ventrally and lateral petals shifting dorsally (Fig. 4).

### CVA reveals differences among all treatments

CV3 slightly separates TRV2-E from the other groups, which could be capturing natural variation in ventral petal length among sampled plants, given the subtle shape change and overlap among the data. CV1 and CV2, on the other hand, describe a shift toward a more radially symmetrical flower. Untreated and mock-treated (TRV2-E) plants overlap in CV1, with an increasing shift toward the positive direction separating *FgCYC2A* + *FgANS* from *FgANS* and *FgCYC2A* knockdowns. *FgCYC2A* and *FgANS* knockdowns are the furthest in the positive direction, with both groups having flowers with wider corolla tubes and lateral petals shifting in the dorsal direction. In both *FgCYC2A* and *FgANS* knockdowns, dorsal petal landmarks are shifted ventrally; however, we hypothesize that different morphological shifts are forming this result. In *FgCYC2A* knockdowns, dorsal petals move ventrally by increasing the angle between the dorsal petals, as seen in CV2 and in pairwise comparisons (Figs. 3 and 4). In *FgANS* knockdowns, the dorsal petal landmarks appear to move downward because the dorsal petals are shorter (see Fig. 4). Furthermore, the CVA plots indicate that shape differences in *FgANS* and *FgCYC2A* knockdowns are opposing each other in CV1, as the double knockdowns are not shifted as much as either single knockdown. This is likely because dorsal petals of *FgCYC2A* knockdowns are longer and farther from the axis of symmetry, while those of *FgANS* knockdowns are shorter and closer to the axis of symmetry, suggesting a possible direct opposition between length and placement of dorsal petals.

These data indicate that *ANS* could confer subtle changes in floral morphology, especially since CV1 and CV2 together separate *FgANS* knockdowns from all other treatments. Traits such as the concentration of *ANS* pigments in flowers and the width of the corolla tube opening have been shown to be correlated with pollinator preference [91, 92], suggesting that *ANS* could be involved in other, non-pigment traits related to pollination. It is also possible that subtle shape shifts in *FgANS* knockdowns could be the result of energy or resource allocation, since the thick anthocyanin guides are no longer being generated. In *F. graciliflora*, *ANS* is utilized differently in the lateral and ventral petals, indicating it is being regulated asymmetrically in this group. Regardless, because *ANS* is used as a reporter gene to discern successful inoculation and has previously been used in VIGS [3, 32, 37, 49, 50], researchers should be aware of the potentiality for small shifts in growth.

### Procrustes distance and centroid size

Using geometric morphometrics has allowed us to quantify the Procrustes distance among untreated flowers and each of the experimental treatments (Fig. 5). As expected, untreated flowers were the most similar in shape to the mock-treated controls, while the greatest shape difference occurred between untreated and *FgCYC2A* knockdowns flowers. These types of data could be used to test hypotheses about the efficiency of protocol design. For instance, VIGS protocols for double gene knockdowns typically include both genes on a single TRV2 construct [32, 36, 37, 93], although when using the *Barley Stripe Mosaic Virus*, researchers have successfully inoculated and downregulated genes with fragments on two separate TRV2 constructs [94, 95]. Our double knockdowns of *FgANS* and *FgCYC2A* utilised the latter protocol, as the initial aim was to utilise *FgANS* to visualise successful downregulation. In the double knockdowns, TRV2-*FgANS* and TRV2-*FgCYC2A* were added in a (1: 1) ratio, resulting in only half the concentration of each vector being added compared to single knockdowns. Our results show a stronger phenotype in the single *FgCYC2A* knockdowns when compared to the double knockdowns, with the shape distance between each treatment and the untreated flowers being more than 1.5 times larger (around 1.66) in the single knockdowns. Examining average shape pairwise comparisons (Fig. 4), the double knockdowns show a similar direction of landmark movement as the single *FgCYC2A* knockdowns, but they are not as shifted. Future experiments could include examining shape distance following a double knockdown that utilises a single TRV2 vector containing portions of both *FgANS* and *FgCYC2A*. Calculating Procrustes distances between the control and different treatments in a given experiment could be used to compare efficiency among protocols.

In addition to overall shape distance, the centroid size varied among the treatments as well. *FgCYC2A* knockdowns had a significantly smaller centroid size then all the other treatments, perhaps due to a more radially symmetrical flower and/or by the development of smaller flowers overall. *FgANS* knockdowns, on the other hand, had a centroid size that was significantly greater than the untreated controls, perhaps due to a more elongated flower and/or by the development of larger flowers overall. The double knockdowns are more similar to *FgANS* knockdowns than to *FgCYC2A* knockdowns supporting the hypothesis that the loss of anthocyanin pigment may mean that more energy is available for growth, resulting in slightly larger flowers.

## Conclusions

### The power of coupling VIGS with geometric morphometrics

Using VIGS on non-model species, such as *Fedia graciliflora*, can serve to provide fast, useful information about gene function provided that the morphological changes can be quantified and clearly analysed. While geometric morphometrics has long been used in animal studies, there is an increasing interest in using it to analyse flower and leaf shape (e.g., [3, 14, 96–99]), in taxonomic (e.g., [13, 100]), in ecological (e.g., [101, 102]), and in evolutionary contexts (e.g., [3, 103–105]). Here, for the first time, we illustrate how geometric morphometrics can be effectively used to quantify the phenotypic effects of knocking down a single CYC2 paralog, *FgCYC2A*, as well as the reporter gene, *FgANS*. Future studies could examine the specific components of shape that are effected by each *CYC*-like paralog to better determine (1) whether different paralogs exhibit partially redundant function; and (2) how the cumulative effect of the paralogs adds together to form increasing complexity in shape. This opens a wide range of new potential applications to further understand the genetics and developmental origins of morphological variation in flowers, particularly in the context of symmetry and asymmetry that is hypothesised to have played an important role in the adaptive diversification of angiosperms.

## Additional files

Additional file 1: Variation in *Fedia graciliflora ANTHOCYANIDIN SYNTHASE* (*FgANS*) knockdown flowers. (a) Weakly downregulated phenotype. (b) Medium downregulated phenotype. (c) Strongly downregulated phenotype. (Scale bar, 2 mm). (EPS 13471 kb)

Additional file 2: Primers used in cloning, RACE, and qPCR of *FgANS* and *FgCYC2A*. Location of cloning and qPCR primers is shown in Additional file 3. *, designates degenerate primers. (XLS 29 kb)

Additional file 3: Virus-induced gene silencing (VIGS) vector fragment and TRV construct schematics. (a) Schematics of *Fedia graciliflora ANTHOCYANIDIN SYNTHASE* (*FgANS*) and *F. graciliflora CYCLOIDEA2A* (*FgCYC2A*) cDNAs including primer positions. Images are not drawn to scale. (b) Schematic of TRV1 and TRV2 constructs. LB = left border, RB = right border, RdRp = RNA-dependent RNA polymerase, MP = movement protein, 16 K = 16Kd protein, Rz = self-cleaving ribozyme, NOst = NOS terminator, CP = coat protein, MCS = multiple cloning site. (EPS 1521 kb)

Additional file 4: Quantitative real-time PCR (qPCR) analysis of *FgANS* and *FgCYC2A* in untreated samples and knockdown treatments. (a) qPCR of *FgANS* showing biological replicates from untreated samples (*N* = 4) compared to those from *FgANS* (*N* = 6) and *FgCYC2A + FgANS* (*N* = 3) knockdown flower buds. (b) qPCR of *FgCYC2A* showing biological replicates from untreated samples (*N* = 3) compared to those from *FgCYC2A* (*N* = 3) and *FgCYC2A* + *FgANS* (*N* = 3) knockdown flower buds. (EPS 1055 kb)

Additional file 5: ANOVA for centroid size comparison between the untreated samples and the treatments followed by Tukey’s HSD test for pairwise differences. Tukey multiple comparisons of means with 95% family-wise confidence level and *P*-value adjusted for multiple comparisons. Diff, difference in the observed means; lwr, lower end point of the interval based on the range of the sample means; upr, upper end point; p adj, *P*-value after adjustment for the multiple comparisons. (XLS 62 kb)

Additional file 6: Test for pairwise differences in shape between the untreated samples and the treatments. *P*-values from permutation tests (10,000 permutation rounds) for Procrustes distances among groups are indicated as: ****, *P* < 0.0001; *, *P* < 0.05. (XLS 60 kb)

Additional file 7: Quantitative real-time PCR (qPCR) analysis of *Fedia graciliflora ANTHOCYANIDIN SYNTHASE* (*FgANS*) untreated and mock-treated (TRV2-E) flower buds. No significant difference in expression was observed. (TIFF 7723 kb)

Additional file 8: Quantitative real-time PCR (qPCR) analysis of *F. graciliflora CYCLOIDEA2A* (*FgCYC2A*) untreated and mock-treated (TRV2-E) flower buds. No significant difference in expression was observed. (TIFF 7702 kb)

Additional file 9: ANOVAs for measurement error for shape and size concerning the untreated sample. SS, sum of squares; MS, mean square; Df, degrees of freedom; F, F-value; *P*, *P* value. (XLS 28 kb)

## Abbreviations

‘HSD’: Honest significant difference
ANOVA: Analysis of variance
ANS: *ANTHOCYANIDIN SYNTHASE*
bp: Base pair
C: Celsius
cDNA: Complementary DNA
CV1: Canonical variate axis 1
CV2: Canonical variate axis 2
CV3: Canonical variate axis 3
CVA: Canonical variate analysis
CYC: *CYCLOIDEA*
CYC2A: *CYCLOIDEA2A*
DICH: *DICHOTOMA*
evo-devo: Evolutionary developmental biology
Fg: *Fedia graciliflora*
FgANS: *Fedia graciliflora ANTHOCYANIDIN SYNTHASE*
FgCYC2A: *Fedia graciliflora CYCLOIDEA2A*
GAPDH: *GLYCERALDEHYDE 3-PHOSPHATE DEHYDROGENASE*
GOI: Gene of interest
h: Hour
K: Kelvin
LB: Lysogeny broth
mg L^−1^: Milligrams per liter
MgCl_2_: Magnesium chloride
mL: Milliliter
N: Sample size
ng mL^−1^: Nanograms per milliliter
PCR: Polymerase chain reaction
pTRV1: TRV RNA1 plasmid
pTRV2: TRV RNA2 plasmid
qPCR: Quantitative real-time PCR
TCP: tb1 cycloidea PCF transcription factor family
TRV: Tobacco rattle virus
TRV2-E: Plants inoculated with TRV2 empty vector
TRV2-FgANS: Plants inoculated with TRV2 containing *FgANS* fragment
TRV2-FgCYC2A: Plants inoculated with TRV2 containing *FgCYC2A* fragment
VIGS: Virus-induced gene silencing
W: Watts
μL: Microliter
μM: Micromolar

## References

[1] Chanderbali AS, Berger BA, Howarth DG, Soltis PS, Soltis DE (2016). Evolving ideas on the origin and evolution of flowers: new perspectives in the genomic era. Genetics.

[2] Howarth DG, Dunn MP, Kliman RM (2016). Phylogenetic approach to studying developmental evolution: a model clade approach. Encyclopedia of evolutionary biology.

[3] Wang P, Liao H, Zhang W, Yu X, Zhang R, Shan H (2015). Flexibility in the structure of spiral flowers and its underlying mechanisms. Nat Plants.

[4] Zhang J-S, Zhao J, Zhang S, He C (2014). Efficient gene silencing mediated by tobacco rattle virus in an emerging model plant *Physalis*. PLoS One.

[5] Preston JC, Barnett LL, Kost MA, Oborny NJ, Hileman LC (2014). Optimization of virus-induced gene silencing to facilitate evo-devo studies in the emerging model species *Mimulus guttatus* (Phrymaceae). Ann Mo Bot Gard.

[6] Donoghue MJ, Bell CD, Winkworth RC (2003). The evolution of reproductive characters in Dipsacales. Int J Plant Sci.

[7] Jacobs B, Bell C, Smets E (2010). Fruits and seeds of the *Valeriana* clade (Dipsacales): diversity and evolution. Int J Plant Sci.

[8] Bell CD, Kutschker A, Arroyo MTK (2012). Phylogeny and diversification of Valerianaceae (Dipsacales) in the southern Andes. Mol Phylogenet Evol.

[9] de Enrech Xena N, Mathez J (1990). Revision du genre *Fedia* Gaertn. Emend Moench (Valerianaceae). Nat Monspel Ser Bot.

[10] de Enrech Xena N, Cardona A, Mathez J (1991). Estudio citotaxonómico del género *Fedia* Gaertn. (Valerianaceae). Anales Jard Bot Madrid.

[11] Hidalgo O, Mathez J, Garcia S, Garnatje T, Pellicer J, Vallès J (2010). Genome size study in the Valerianaceae: first results and new hypotheses. J Bot.

[12] Zapata L, Ding J, Willing E-M, Hartwig B, Bezdan D, Jiao W-B (2016). Chromosome-level assembly of *Arabidopsis thaliana* L*er* reveals the extent of translocation and inversion polymorphisms. Proc Natl Acad Sci U S A.

[13] Shipunov AB, Bateman RM (2005). Geometric morphometrics as a tool for understanding *Dactylorhiza* (Orchidaceae) diversity in European Russia. Biol J Linn Soc.

[14] Gomez JM, Perfectti F, Camacho JPM (2006). Natural selection on *Erysimum mediohispanicum* flower shape: insights into the evolution of zygomorphy. Amer Nat.

[15] Citerne H, Jabbour F, Nadot S, Damerval C, Citerne H, Jabbour F, Nadot S, Damerval C. The evolution of floral symmetry. In: Kader J-C, Delseny M, editors. Advances in botanical research: Elsevier Ltd; 2010. p. 85–137.

[16] Endress P (1999). Symmetry in flowers: diversity and evolution. Int J Plant Sci.

[17] Sargent RD (2004). Floral symmetry affects speciation rates in angiosperms. Proc R Soc Lond B.

[18] Neal PR, Dafni A, Giurfa M (1998). Floral symmetry and its role in plant-pollinator systems: terminology, distribution, and hypotheses. Annu Rev Ecol Syst.

[19] Luo D, Carpenter R, Vincent C, Copsey L, Coen E (1996). Origin of floral asymmetry in *Antirrhinum*. Nature.

[20] Howarth DG, Donoghue MJ (2006). Phylogenetic analysis of the “ECE” (*CYC*/*TB1*) clade reveals duplications predating the core eudicots. Proc Natl Acad Sci U S A.

[21] Hileman LC. Trends in flower symmetry evolution revealed through phylogenetic and developmental genetic advances. Phil Trans R Soc B. 2014;369:20130348–8. 10.1098/rstb.2013.0348 PMC4071522 24958922

[22] Berger BA, Thompson V, Lim A, Ricigliano V, Howarth DG. Elaboration of bilateral symmetry across *Knautia macedonica* capitula related to changes in ventral petal expression of *CYCLOIDEA*-like genes. EvoDevo. 2016;7:8. 10.1186/s13227-016-0045-7 PMC4818532 27042288

[23] Feng X, Zhao Z, Tian Z, Xu S, Luo Y, Cai Z (2006). Control of petal shape and floral zygomorphy in *Lotus japonicus*. Proc Natl Acad Sci U S A.

[24] Kim M, Cui M-L, Cubas P, Gillies A, Lee K, Chapman MA (2008). Regulatory genes control a key morphological and ecological trait transferred between species. Science.

[25] Wang Z, Luo Y, Li X, Wang L, Xu S, Yang J (2008). Genetic control of floral zygomorphy in pea (*Pisum sativum* L.). Proc Natl Acad Sci U S A.

[26] Specht CD, Howarth DG (2014). Adaptation in flower form: a comparative evodevo approach. New Phytol.

[27] Howarth DG, Donoghue MJ (2005). Duplications in *CYC*-like genes from Dipsacales correlate with floral form. Int J Plant Sci.

[28] Howarth DG, Martins T, Chimney E, Donoghue MJ. Diversification of *CYCLOIDEA* expression in the evolution of bilateral flower symmetry in Caprifoliaceae and *Lonicera* (Dipsacales). Ann Bot. 2011;107:1521–32. 10.1093/aob/mcr049 PMC3108805 21478175

[29] Di Stilio VS, Kumar RA, Oddone AM, Tolkin TR, Salles P, McCarty K (2010). Virus-induced gene silencing as a tool for comparative functional studies in *Thalictrum*. PLoS One.

[30] Hidalgo O, Bartholmes C, Gleissberg S (2012). Virus-induced gene silencing (VIGS) in *Cysticapnos vesicaria*, a zygomorphic-flowered Papaveraceae (Ranunculales, basal eudicots). Ann Bot.

[31] Galimba KD, Tolkin TR, Sullivan AM, Melzer R, Theissen G, Di Stilio VS (2012). Loss of deeply conserved C-class floral homeotic gene function and C- and E-class protein interaction in a double-flowered ranunculid mutant. Proc Natl Acad Sci U S A.

[32] Gonçalves B, Nougué O, Jabbour F, Ridel C, Morin H, Laufs P (2013). An *APETALA3* homolog controls both petal identity and floral meristem patterning in *Nigella damascena* L. (Ranunculaceae). Plant J.

[33] Hsieh M-H, Pan Z-J, Lai P-H, Lu H-C, Yeh H-H, Hsu C-C (2013). Virus-induced gene silencing unravels multiple transcription factors involved in floral growth and development in *Phalaenopsis* orchids. J Exp Bot.

[34] Pan Z-J, Chen Y-Y, Du J-S, Chen Y-Y, Chung M-C, Tsai W-C (2014). Flower development of *Phalaenopsis* orchid involves functionally divergent *SEPALLATA*-like genes. New Phytol.

[35] Sung YC, Lin CP, Chen JC (2014). Optimization of virus-induced gene silencing in *Catharanthus roseus*. Plant Pathol.

[36] Zhang S, Zhang J-S, Zhao J, He C (2015). Distinct subfunctionalization and neofunctionalization of the B-class MADS-box genes in *Physalis floridana*. Planta.

[37] Gould B, Kramer EM (2007). Virus-induced gene silencing as a tool for functional analyses in the emerging model plant *Aquilegia* (columbine, Ranunculaceae). Plant Methods.

[38] Velásquez AC, Chakravarthy S, Martin GB. Virus-induced gene silencing (VIGS) in *Nicotiana benthamiana* and tomato. J Vis Exp. 2009:e1292. 10.3791/1292 PMC2795700 19516240

[39] Becker A, Lange M (2010). VIGS – genomics goes functional. Trends Plant Sci.

[40] Burch-Smith TM, Anderson JC, Martin GB, Dinesh-Kumar SP (2004). Applications and advantages of virus-induced gene silencing for gene function studies in plants. Plant J.

[41] Unver T, Budak H (2009). Virus-induced gene silencing, a post transcriptional gene silencing method. Int J Plant Genomics.

[42] Bond DM, Baulcombe DC (2015). Epigenetic transitions leading to heritable, RNA-mediated de novo silencing in *Arabidopsis thaliana*. Proc Natl Acad Sci U S A.

[43] Kim BM, Inaba J-I, Masuta C (2011). Virus induced gene silencing in *Antirrhinum majus* using the *Cucumber Mosaic Virus* vector: functional analysis of the *AINTEGUMENTA* (*Am*-*ANT*) gene of *A. majus*. Hort Environ Biotechnol.

[44] Smaczniak C, Immink RGH, Angenent GC, Kaufmann K (2012). Developmental and evolutionary diversity of plant MADS-domain factors: insights from recent studies. Development.

[45] Senthil-Kumar M, Mysore KS (2011). Virus-induced gene silencing can persist for more than 2 years and also be transmitted to progeny seedlings in *Nicotiana benthamiana* and tomato. Plant Biotech J.

[46] Meins F, Si-Ammour A, Blevins T (2005). RNA silencing systems and their relevance to plant development. Annu Rev Cell Dev Biol.

[47] Kumar P, Pandit SS, Baldwin IT (2012). Tobacco rattle virus vector: a rapid and transient means of silencing *Manduca sexta* genes by plant mediated RNA interference. PLoS One.

[48] Jiang Y, Ye S, Wang L, Duan Y, Lu W, Liu H (2013). Heterologous gene silencing induced by tobacco rattle virus (TRV) is efficient for pursuing functional genomics studies in woody plants. Plant Cell Tissue Organ Cult.

[49] Sharma B, Guo C, Kong H, Kramer EM (2011). Petal-specific subfunctionalization of an *APETALA3* paralog in the Ranunculales and its implications for petal evolution. New Phytol.

[50] Sharma B, Kramer E (2012). Sub- and neo-functionalization of *APETALA3* paralogs have contributed to the evolution of novel floral organ identity in *Aquilegia* (columbine, Ranunculaceae). New Phytol.

[51] Campanella JJ, Smalley JV, Dempsey ME (2014). A phylogenetic examination of the primary anthocyanin production pathway of the Plantae. Bot Stud.

[52] Slice DE (2007). Geometric morphometrics. Annu Rev Anthropol.

[53] Zelditch ML, Swiderski DL, Sheets HD (2012). Geometric morphometrics for biologists: a primer.

[54] Adams DC, Rohlf FJ, Slice DE (2013). A field comes of age: geometric morphometrics in the 21st century. Hystrix.

[55] Klingenberg CP, McIntyre GS (1998). Geometric morphometrics of developmental instability: analyzing patterns of fluctuating asymmetry with Procrustes methods. Evolution.

[56] Mardia KV, Bookstein FL, Moreton IJ (2000). Statistical assessment of bilateral symmetry of shapes. Biometrika.

[57] Kent JT, Mardia KV (2001). Shape, Procrustes tangent projections and bilateral symmetry. Biometrika.

[58] Klingenberg CP, Barluenga M, Meyer A (2002). Shape analysis of symmetric structures: quantifying variation among individuals and asymmetry. Evolution.

[59] Savriama Y, Neustupa J, Klingenberg CP (2010). Geometric morphometrics of symmetry and allometry in *Micrasterias rotata* (Zygnemophyceae, Viridiplantae). Nova Hedwig Beih.

[60] Savriama Y, Klingenberg CP (2011). Beyond bilateral symmetry: geometric morphometric methods for any type of symmetry. BMC Evol Biol.

[61] Savriama Y, Gómez JM, Perfectti F, Klingenberg CP (2012). Geometric morphometrics of corolla shape: dissecting components of symmetric and asymmetric variation in *Erysimum mediohispanicum* (Brassicaceae). New Phytol.

[62] Ratcliff F, Martin-Hernandez AM, Baulcombe DC (2001). Tobacco rattle virus as a vector for analysis of gene function by silencing. Plant J.

[63] Liu Y, Schiff M, Marathe R, Dinesh-Kumar SP. *Tobacco Rar1*, *EDS1* and *NPR1*/*NIM1* like genes are required for *N*-mediated resistance to tobacco mosaic virus. Plant J. 2002;30:415–29. 10.1046/j.1365-313x.2002.01297.x 12028572

[64] Livak KJ, Schmittgen TD (2001). Analysis of relative gene expression data using real-time quantitative PCR and the 2−ΔΔCT method. Methods.

[65] Dryden IL, Mardia KV. Statistical shape analysis. Chichester: Wiley; 1998.

[66] Adams DC, Rohlf FJ, Slice DE (2004). Geometric morphometrics: ten years of progress following the “revolution”. Ital J Zool.

[67] Rohlf FJ (2015). The tps series of software. Hystrix.

[68] Rohlf FJ, Slice D (1990). Extensions of the Procrustes method for the optimal superimposition of landmarks. Syst Biol.

[69] Goodall C (1991). Procrustes methods in the statistical analysis of shape. J R Statist Soc B.

[70] Slice DE (2001). Landmark coordinates aligned by Procrustes analysis do not lie in Kendall's shape space. Syst Biol.

[71] Leamy L (1984). Morphometric studies in inbred and hybrid house mice. V. Directional and fluctuating asymmetry. Amer Nat.

[72] Palmer AR, Strobeck C (1986). Fluctuating asymmetry: measurement, analysis, patterns. Annu Rev Ecol Syst.

[73] Slice DE, Bookstein FL, Marcus LF, Rohlf FJ, Marcus LF, Corti M, Loy A, GJP N, Slice DE (1996). Appendix I: a glossary for geometric morphometrics. Advances in Morphometrics. NATO ASI SERIES a LIFE SCIENCES.

[74] Rohlf FJ (1999). Shape statistics: Procrustes superimpositions and tangent spaces. J Classif.

[75] R Development Core Team (2016). R: a language and environment for statistical computing.

[76] Klingenberg CP (2016). Size, shape, and form: concepts of allometry in geometric morphometrics. Dev Genes Evol.

[77] Drake AG, Klingenberg CP (2008). The pace of morphological change: historical transformation of skull shape in St Bernard dogs. Proc R Soc B.

[78] Albrecht GH (1980). Multivariate analysis and the study of form, with special reference to canonical variate analysis. Amer Zool.

[79] Campbell NA, Atchley WR (1981). The geometry of canonical variate analysis. Syst Biol.

[80] Cruz-Castillo JG, Ganeshanandam S, MacKay BR, Lawes GS, Lawoko CRO, Woolley DJ (1994). Applications of canonical discriminant analysis in horticultural research. Hortscience.

[81] Fernandez-Mazuecos M, Blanco-Pastor JL, Gomez JM, Vargas P (2013). Corolla morphology influences diversification rates in bifid toadflaxes (*Linaria* sect. *Versicolores*). Ann Bot.

[82] Medel R, Botto-Mahan C, Kalin-Arroyo M (2003). Pollinator-mediated selection on the nectar guide phenotype in the Andean monkey flower, *Mimulus luteus*. Ecology.

[83] Good P (2013). Permutation tests: a practical guide to resampling methods for testing hypotheses.

[84] Klingenberg CP (2011). MorphoJ: an integrated software package for geometric morphometrics. Mol Ecol Resour.

[85] Liu YL, Schiff M, Dinesh-Kumar SP (2002). Virus-induced gene silencing in tomato. Plant J.

[86] Prud'homme B, Gompel N, Rokas A, Kassner VA, Williams TM, Yeh S-D (2006). Repeated morphological evolution through *cis*-regulatory changes in a pleiotropic gene. Nature.

[87] Gao Q, Tao JH, Yan D, Wang YZ, Li ZY. Expression differentiation of *CYC*-like floral symmetry genes correlated with their protein sequence divergence in *Chirita heterotricha* (Gesneriaceae). Dev Genes Evol. 2008;218:341–51. 10.1007/s00427-008-0227-y 18592267

[88] Zhou XR, Wang YZ, Smith JF, Chen R. Altered expression patterns of TCP and MYB genes relating to the floral developmental transition from initial zygomorphy to actinomorphy in *Bournea* (Gesneriaceae). New Phytol. 2008;178:532–43. 10.1111/j.1469-8137.2008.02384.x 18312540

[89] Song CF, Lin QB, Liang RH, Wang YZ. Expressions of ECE-CYC2 clade genes relating to abortion of both dorsal and ventral stamens in *Opithandra* (Gesneriaceae). BMC Evol Biol. 2009;9:244. 10.1186/1471-2148-9-244 PMC2763874 19811633

[90] Preston JC, Kost MA, Hileman LC (2009). Conservation and diversification of the symmetry developmental program among close relatives of snapdragon with divergent floral morphologies. New Phytol.

[91] Schemske D, Bradshaw H. Pollinator preference and the evolution of floral traits in monkeyflowers (*Mimulus*). Proc Natl Acad Sci USA. 1999;96:11910. 10.1073/pnas.96.21.11910 PMC18386 10518550

[92] Nakazato T, Rieseberg LH, Wood TE (2013). The genetic basis of speciation in the *Giliopsis* lineage of *Ipomopsis* (Polemoniaceae). Heredity.

[93] Kramer EM, Holappa L, Gould B, Jaramillo MA, Setnikov D, Santiago PM (2007). Elaboration of B gene function to include the identity of novel floral organs in the lower Eudicot *Aquilegia*. Plant Cell.

[94] Cakir C, Scofield S (2008). Evaluating the ability of the barley stripe mosaic virus-induced gene silencing system to simultaneously silence two wheat genes. Cereal Res Commun.

[95] Campbell J, Huang L. Silencing of multiple genes in wheat using barley stripe mosaic virus. J Biotech Res. 2010;2:12-20.

[96] Herrera CM (1993). Selection on floral morphology and environmental determination of fecundity in a hawk moth-pollinated violet. Ecol Monogr.

[97] Frey FM, Robertson A, Bukoski M (2007). A method for quantifying rotational symmetry. New Phytol.

[98] van der Niet T, Zollikofer CPE, de León MSP, Johnson SD, Linder HP (2010). Three-dimensional geometric morphometrics for studying floral shape variation. Trends Plant Sci.

[99] Dalayap RM, Torres MA, Demayo CG. Landmark and outline methods in describing petal, sepal and labellum shapes of the flower of Mokara orchid varieties. Int J Agr Biol. 2011;13:652-58.

[100] Feng X, Wilson Y, Bowers J, Kennaway R, Bangham A, Hannah A (2009). Evolution of allometry in *Antirrhinum*. Plant Cell.

[101] Baranov SG (2014). Use of morphogeometric method for study fluctuating asymmetry in leaves *Tilia cordata* under industrial pollution. Adv Environ Biol.

[102] Vujić V, Avramov S, Tarasjev A (2015). The effects of traffic-related air pollution on the flower morphology of *Iris pumila*-comparison of a polluted city area and the unpolluted Deliblato Sands (nature reserve). Appl Ecol Environ Res.

[103] Albarrán Lara AL, Mendoza Cuenca L, Valencia Avalos S, González Rodríguez A, Oyama K (2010). Leaf fluctuating asymmetry increases with hybridization and introgression between *Quercus magnoliifolia* and *Quercus resinosa* (Fagaceae) through an altitudinal gradient in Mexico. Int J Plant Sci.

[104] Klingenberg CP, Duttke S, Whelan S, Kim M (2011). Developmental plasticity, morphological variation and evolvability: a multilevel analysis of morphometric integration in the shape of compound leaves. J Evol Biol.

[105] Carleial S, Kleunen M, Stift M. Small reductions in corolla size and pollen: ovule ratio, but no changes in flower shape in selfing populations of the north American *Arabidopsis lyrata*. Oecologia. 2017;183:401–13. 10.1007/s00442-016-3773-4 27866292

