# Supplementary figures and images for: Geometric morphometrics reveals shifts in flower shape symmetry and size following gene knockdown of CYCLOIDEA and ANTHOCYANIDIN SYNTHASE
Source: BMC Plant Biol. 2017 Nov 17;17:205. doi: 10.1186/s12870-017-1152-x (PMC5693587; doi:10.1186/s12870-017-1152-x)

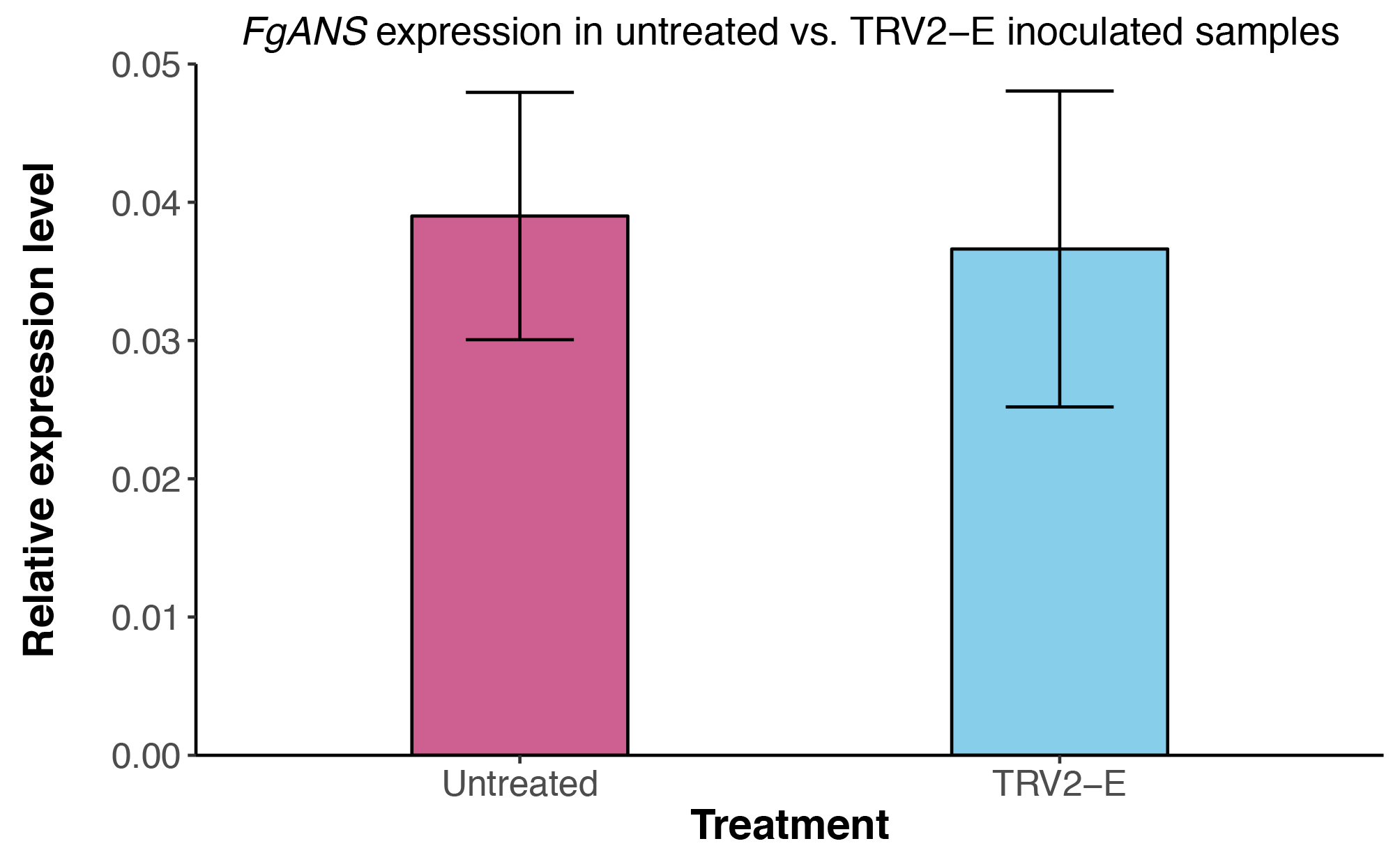

Supplement: Supplementary file 7 — Quantitative real-time PCR (qPCR) analysis of Fedia graciliflora ANTHOCYANIDIN SYNTHASE (FgANS) untreated and mock-treated (TRV2-E) flower buds. No significant difference in expression was observed. (TIFF 7723 kb) [file 12870_2017_1152_MOESM7_ESM.tif]

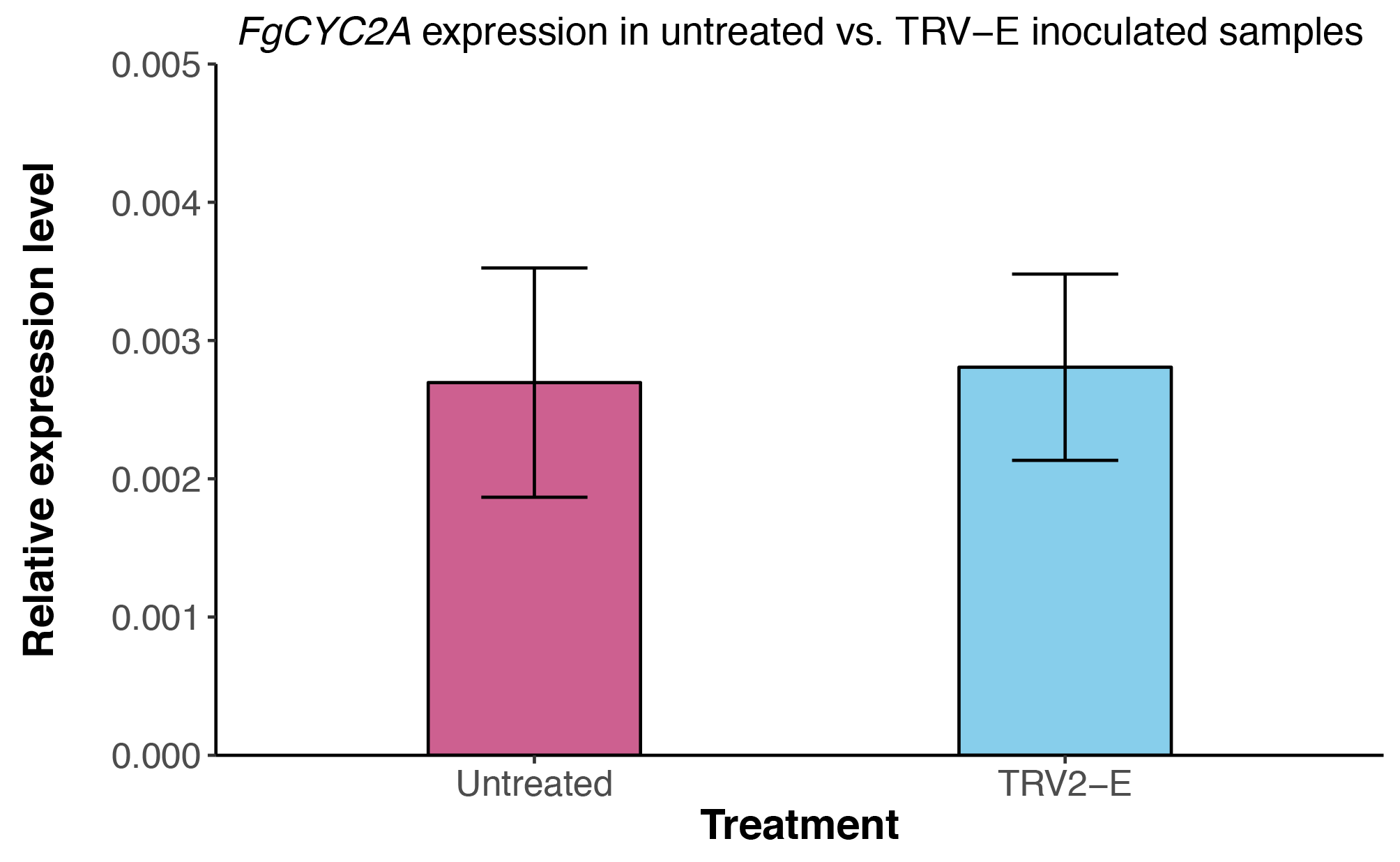

Supplement: Supplementary file 8 — Quantitative real-time PCR (qPCR) analysis of F. graciliflora CYCLOIDEA2A (FgCYC2A) untreated and mock-treated (TRV2-E) flower buds. No significant difference in expression was observed. (TIFF 7702 kb) [file 12870_2017_1152_MOESM8_ESM.tif]
